# Chemotaxonomy, an Efficient Tool for Medicinal Plant Identification: Current Trends and Limitations

**DOI:** 10.3390/plants14142234

**Published:** 2025-07-19

**Authors:** Adnan Amin, SeonJoo Park

**Affiliations:** Department of Life Sciences, Yeungnam University, Gyeongsan 38541, Republic of Korea; adnan.amin@yu.ac.kr

**Keywords:** secondary metabolites, diversity, omics data, artificial intelligence, DNA barcoding

## Abstract

This review highlights the critical role of chemotaxonomy in the identification, authentication, and discovery of bioactive compounds in medicinal plants. By analyzing secondary metabolites using techniques like UV spectroscopy, FTIR, HPLC, GC-MS, NMR, LC-MS-Qtof, and MALDI-TOF MS, chemotaxonomy ensures accurate plant identification, supporting the safe and effective use of plants in herbal medicine. Key secondary metabolites used in chemotaxonomic identification include alkaloids, flavonoids, terpenoids, phenolics, tannins, and plant peptides. Chemotaxonomy also facilitates the discovery of novel compounds with therapeutic potential, contributing to drug development. The integration of chemotaxonomy with genomics and proteomics allows a deeper understanding of plant biosynthesis and the mechanisms behind bioactive compound production. However, challenges due to variability in metabolite profiles and the lack of standardized methods remain, and future research should focus on developing global databases, improving standardization, and incorporating artificial intelligence and machine learning to enhance plant identification and bioactive compound discovery. The integration of chemotaxonomy with personalized medicine offers the potential to tailor plant-based therapies to individual genetic profiles, advancing targeted treatments. This review underscores chemotaxonomy’s importance in bridging traditional knowledge and modern science, offering sustainable solutions for medicinal plant use and drug development.

## 1. Introduction

In plant sciences, “Taxonomy,” the identification, classification, and naming of plants based on shared characteristics and evolutionary relationships, is considered a foundational field of study [1,2]. Taxonomy is crucial in the conservation of endangered species, as identifying and classifying species accurately is necessary for establishing conservation priorities and protecting biodiversity [3]. In the field of medicine, proper plant identification is vital for ensuring the efficacy and safety of plant-derived medicines [4], and taxonomic classification based on secondary metabolites (alkaloids, flavonoids, terpenoids, etc.) aids in discovering new bioactive compounds for drug development [5]. Similarly, in agricultural sciences, taxonomy supports crop improvement by identifying and classifying economically important plants [6], helping to develop pest-resistant varieties and optimize cultivation practices. With the advent of molecular techniques like DNA barcoding, the accuracy and speed of plant identification have greatly improved, allowing precise classification and even parsing species complexes and identifying cryptic species [7,8].

Classical plant taxonomy emphasizes the use of stable morphological characters that are minimally influenced by environmental factors for taxonomic distinction, excluding traits with high phenotypic plasticity [9]. However, in practice, especially when dealing with commercialized plant materials often presented as ground or fragmented forms lacking key diagnostic organs, reliable identification using macroscopic morphology becomes challenging [10]. In these cases, microscopic anatomical features, molecular markers (DNA-based methods), and chemical profiling can provide more robust and accurate means of identification [11], particularly for medicinal plants, where precise authentication is critical. Palynology, which uses the size, shape, and surface texture of pollen grains to identify plant species, is another valuable method, especially in ecological and archaeological contexts [12]. Molecular methods, such as DNA barcoding, have revolutionized plant identification. By providing a genetic “barcode” using markers like *rbcL* and *matK*, precise species identification is possible even in cases where morphological features are absent or ambiguous [13]. Additionally, metabolomics, which uses advanced technologies like liquid chromatography–mass spectrometry (LC-MS) and nuclear magnetic resonance (NMR) to profile the entire metabolome, provides in-depth insights into plant chemistry, further aiding species differentiation [14,15]. Moreover, artificial intelligence (AI) and machine learning (ML) are increasingly being integrated with traditional methods, enhancing the efficiency of plant classification by automating data analysis and detecting complex patterns in chemical and molecular datasets [16]. Through these advances, chemotaxonomy has become important, since this science uses secondary metabolites like flavonoids, alkaloids, terpenoids, and phenolic compounds analyzed through techniques such as high-performance liquid chromatography (HPLC), gas chromatography–mass spectrometry (GC-MS)**,** and LC-MS with quadrupole time of flight (LC-MS-Qtof) [17]. Such high-throughput techniques are helpful to distinguish species based on chemical profile, thus offering high accuracy in cases of closely related or cryptic species [18]. Interestingly, multiple plant identification techniques, including DNA barcoding, AI, and chemotaxonomy, are increasingly being integrated, with such protocols often referred to as hybrid methods [19].

Chemotaxonomy is an old but now rapidly evolving field that plays a significant role in plant classification and identification [20]. Secondary metabolites are often unique to plant species and provide valuable insights into their evolutionary relationships [21,22]. This integration of morphological and chemical traits allows for more accurate and reliable plant identification, which is especially critical in the study of medicinal plants. Chemotaxonomy has witnessed significant advances in recent years, particularly with the integration of molecular biology techniques. For instance, DNA barcoding has become a popular method for plant identification, offering a molecular-marker-based approach that complements chemical profiling [23]. Metabolomics, through the comprehensiveness of the catalog of metabolites, can be used for specific plant tissues and has also enhanced the capabilities of chemotaxonomy [24]. Additionally, multivariate analysis techniques, such as principal component analysis (PCA) and cluster analysis (CA), have enabled researchers to better correlate chemical data with taxonomic information, improving the accuracy of plant classification [25]. Furthermore, the integration of bioinformatic tools and databases has facilitated the analysis and storage of large volumes of chemotaxonomic data, making it easier to access and compare plant profiles from different regions and studies [20,26].

The objective of this review is to provide an in-depth exploration of the current trends in chemotaxonomy, particularly in the context of medicinal plant identification. It highlights recent technological advances in chemotaxonomy, including the latest analytical techniques and molecular methods used to study plant chemical composition. It also discusses the integration of chemotaxonomy with traditional taxonomy and molecular biology to improve the accuracy and reliability of plant identification.

## 2. Concept of Chemotaxonomy and Medicinal Plant Identification

**Figure 1:**
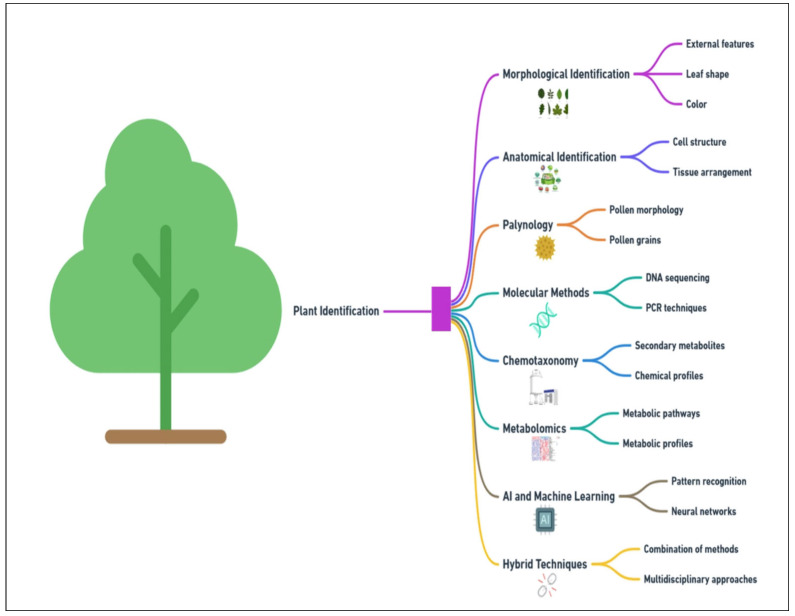
Taxonomical identification methods used by researchers in previous and current eras.

Chemotaxonomy is a discipline that not only utilizes the chemical characteristics of a plant to classify plants but also distinguishes between closely related species [20] and differentiates intraspecific taxa such as subspecies and varieties [27]. This makes it an essential and nuanced tool in plant identification and classification. Unlike traditional morphological classification, chemotaxonomy focuses on the chemical compounds found within a plant. Secondary metabolites are often characteristic of specific plant species and families, making them valuable for taxonomic classification [28]. However, their levels and presence can vary significantly depending on factors such as plant developmental stage, organ type, harvest time, and environmental conditions [29]. Therefore, while secondary metabolites can complement morphological traits in classification, their variability should be carefully considered during chemotaxonomic studies. This technique is often used in conjunction with traditional morphological methods, and together, they provide a deeper, molecular-level understanding of plant diversity. However, by analyzing their chemical profiles, chemotaxonomy can uncover subtle differences between species that are otherwise unobservable (Figure 1).

## 3. Primary and Secondary Metabolites in Medicinal Plants

In medicinal plants, primary metabolites (i.e., carbohydrates, amino acids, proteins, and fatty acids) are inherently essential to the plant’s basic growth and cellular processes [30]. They play key roles in energy production, structural functions, and cell division within the plant, and are crucial to overall plant health and survival [31]. Secondary metabolites, on the other hand, are considered non-essential compounds not directly involved in basic plant functions but serve key ecological roles in, for example, defense against herbivores, pathogens, and environmental stressors [32]. These compounds include an array of diverse classes like alkaloids, flavonoids, terpenoids, phenolic acids, glycosides, etc., which are the primary pharmacologically active compounds in medicinal plants [33] (Figure S1). These pharmacological activities include anti-inflammatory, antimicrobial, anticancer, antidiabetic, antiviral, and antioxidant activities, making them critical in the development of new therapeutic agents [34,35] (Table 1, Figure S2).

## 4. Chemotaxonomy vs. Traditional Morphological Taxonomy

Morphological plant identification that is based on physical plant features including leaf shape, flower color, fruit type, and plant size, has been a foundation of plant taxonomy for centuries [39]. While this method provides an easily accessible and non-invasive approach, environmental factors, phenotypic plasticity, and developmental stage variations are key limits that can affect accuracy [40,41]. Chemotaxonomy, on the other hand, relies on the chemical composition of plants, particularly the secondary metabolites, which are more stable and consistent traits in plants [20,42]. Additionally, chemotaxonomy can identify cryptic species that may appear morphologically similar but differ in chemical composition [43]. Chemotaxonomy can therefore be considered as a “complementing tool” that can facilitate robust plant identification.

Combining morphological characteristics with chemical analysis offers a more comprehensive understanding of plant relationships. Chemotaxonomy thus has the potential to enhance the accuracy of plant classification, particularly when used in combination with molecular tools, such as DNA barcoding [9]. Together, these methods can offer a more complete and precise plant identification system, ensuring that medicinal plants are accurately classified and effectively utilized for therapeutic purposes (Table 2).

## 5. Applications of Chemotaxonomy in the Herbal and Medicinal Plant Sciences

**Figure 2:**
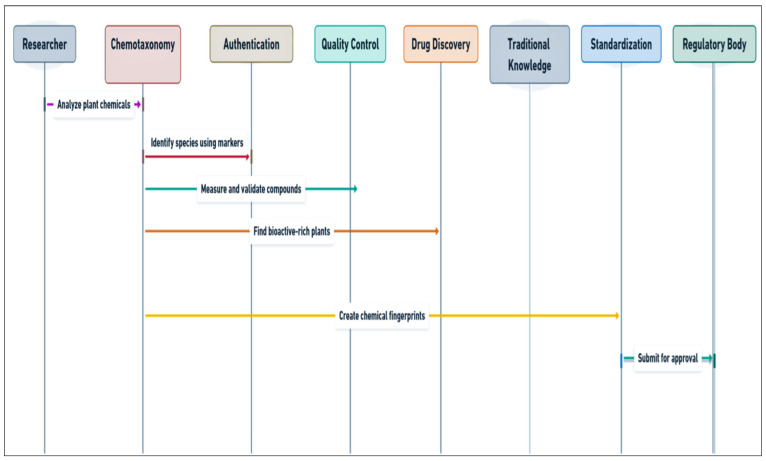
Applications of chemotaxonomy in herbal and medicinal plant sciences.

The primary advantage of chemotaxonomy in medicinal plant identification is its ability to more precisely and reliably distinguish between plant species. As mentioned above, many plants appear similar in terms of their morphology but differ significantly in their chemical composition. This makes chemotaxonomy a powerful tool for identifying plants with unique medicinal properties, as the chemical profile is often directly linked to the plant’s therapeutic potential and ethnopharmacological relevance [47]. Additionally, by focusing on chemical markers, chemotaxonomy allows researchers to identify plants in situations where fresh or whole plants are unavailable, e.g., in cases of adulteration and substitution, which is essential for ensuring the correct plant species is used in herbal medicines and pharmaceuticals [48]. Moreover, chemotaxonomy supports the standardization and quality control of herbal products by providing reproducible chemical markers that act as benchmarks for authentication [49]. This ensures batch-to-batch consistency, which is critical for the efficacy and safety of herbal medicines. Furthermore, it also facilitates regulatory compliance by enabling manufacturers to present validated phytochemical profiles of their raw materials and final products [50] (Figure 2).

## 6. Analytical Methods Used for Compound Identification

Chemotaxonomy involves both qualitative and quantitative analyses of compounds to establish relationships between plant species. In chemotaxonomy, the identification of secondary metabolites is typically carried out using analytical techniques such as HPLC, mass spectrometry (MS, including gas chromatography–MS (GC-MS)], LC-MS-Qtof, matrix-assisted laser desorption/ionization time-of-flight MS (MALDI-TOF MS)), UV and infrared (Fourier transform infrared), and NMR. These techniques allow the efficient separation, identification, and quantification of plant metabolites, providing a chemical “fingerprint” that is unique to each species (Table 3).

### 6.1. HPLC

High-performance liquid chromatography has become indispensable in chemotaxonomy. Its versatility, high resolution, and reproducibility make it ideal for the analysis of complex plant samples containing a wide variety of chemical compounds [56]. Researchers have been applying HPLC to chemotaxonomic identification within plant families for decades, and almost all plant families have been extensively studied using HPLC. Here are a few examples: The Fabaceae family, which includes a large number of economically and medicinally important plants, has been the subject of several such studies. Here, HPLC has been used to identify flavonoids and alkaloids, which have become key chemotaxonomic markers [57]. Similarly, within the genera *Medicago* (alfalfa) and *Cicer* (chickpea), HPLC has been used to profile flavonoids such as quercetin, kaempferol, and their derivatives [58]. These compounds help differentiate species within these genera, as the presence and concentration of specific flavonoids vary between species. Additionally, the alkaloid profiling of *Lupinus* (lupine) species has been investigated using HPLC, identifying compounds such as quinolizidine alkaloids, which are characteristic of this genus [59].

Similarly, chemical identification in the family Solanaceae has been extensively performed using HPLC, with the main focus on alkaloids and other bioactive compounds. In *Capsicum* (chili pepper) species, for instance, the compound responsible for their signature heat, flavor, and pungency, capsaicin, was identified and characterized using HPLC [60]. Similarly, nicotine, the alkaloid that serves as a chemotaxonomic marker for *Nicotiana tabacum* (tobacco) within the family, was also identified using HPLC [61].

Chemotaxonomic identification based on HPLC has also been used in many other plant groups including, the family Asteraceae, which includes well-known medicinal plants, such as *Artemisia annua* (used for malaria treatment) and *Echinacea purpurea* (used for immune support). Flavonoids, sesquiterpenes, and alkaloids have been extensively profiled using HPLC [62,63]. The family Rutaceae (most notable for containing the genus *Citrus*) is another plant group that has been widely studied using HPLC. Citrus fruits, including oranges (*Citrus sinensis*) and lemons (*Citrus limon*), are known for their content of flavonoids and terpenoids, especially hesperidin and narirutin, and these serve as important chemotaxonomic markers within this family [64]. Additionally, detailed data is available on plants of the Rubiaceae family, especially *Coffea arabica* (coffee), which contains extensive concentrations of caffeine, chlorogenic acids, and other alkaloids that are critical for distinguishing between *Coffea* species [65]. More recently, the integration of HPLC with chemometric techniques, like PCA and CA, has further enhanced plant classification. For example, *Coffea* species were differentiated from each other based on their alkaloid and flavonoid content using HPLC and PCA [66]. Similarly, using analyses combining HPLC data with chemometrics, essential oil and secondary metabolite profiles helped define *Coriandrum sativum* (coriander) and *Carum carvi* (caraway) as species [67]. Traditional HPLC mainly utilizes retention time and UV-Vis spectral fingerprints for compound analysis. Advanced systems equipped with photodiode array (PDA) detectors incorporate UV-Vis spectral libraries, allowing tentative compound identification through spectral matching against reference databases (e.g., Agilent OpenLab, ChemStation) [68,69,70]. However, due to overlapping UV absorption profiles among structurally related compounds, identification based solely on UV-Vis spectra often lacks specificity and requires complementary techniques for confirmation.

### 6.2. GC-MS

Gas chromatography–mass spectrometry (GC-MS) is an important technique used for the analysis of volatile compounds. It is widely applied in the study of essential oils containing terpenoids, fatty acids, and polyphenolics, which are responsible for the medicinal properties of many plants [71]. The use of GC-MS in plant chemotaxonomy dates back to the 1970s and 1980s, and this technique plays a key role in chemotaxonomy of volatile compounds.

For several years, GC-Ms has been used for component analysis in a number of diverse plant families. For instance, the Lamiaceae family, which is often referred to as the “mint family,” is well known for its “aromatic” qualities. It includes well-known species like *Mentha piperita* (mint) and genera like *Thymus* (thyme) and *Ocimum* (basil), and it has been extensively studied using GCMS [72]. The *Mentha* species have been classified based on their high concentrations of menthol and other terpenoids using GCMS [73]. Similarly, *Thymus vulgaris* (thyme) mainly contains high concentrations of thymol and carvacrol, which are key components of its essential oil [74]. Thymol and carvacrol are considered as valuable chemotaxonomic markers of similar kinds of compounds that can be helpful in assessing the quality of medicinal herbs.

Another notable application of GC-MS is its use in the analysis of *Apiaceae*, which includes aromatic and medicinal plants like *Carum carvi* (caraway) and *Co. sativum* (coriander). GC-MS has been widely used to identify the volatile compounds in the essential oils of these plants, such as carvone, limonene, and linalool, which are characteristic of Ca. *carvi* and *Co. sativum* [67,75]. These compounds thus serve as chemotaxonomic markers, aiding in the differentiation of species within the *Apiaceae* family. Similarly, *Citrus* species of the Rutaceae family have been studied using GC-MS, with limonene and other terpenes identified as key chemotaxonomic markers for species identification [76].

More recently, GC-MS has been combined with chemometrics to enhance plant species delineation based on chemical profiles [77]. The integration of multivariate statistical techniques, such as PC and CA, with GC-MS data has improved our ability to differentiate closely related plant species, detecting even subtle variations in their chemical profiles [78] Additionally, combining GC-MS and LC-MS allows the development of a complete chemical fingerprint of a plant, helping researchers identify the key compounds responsible for its therapeutic properties [79].

### 6.3. LC-MS-Qtof

Liquid chromatography–mass spectrometry with quadrupole time of flight is an advanced analytical tool in chemotaxonomy that enables the precise identification of a plant species based on their secondary metabolites [80]. This high-resolution technique combines the separation capabilities of liquid chromatography with the accuracy of time-of-flight mass spectrometry, making it ideal for producing plant chemical profiles. The application of LC-MS-Qtof in chemotaxonomy began in the early 2000s, at which time it represented a new approach. Early studies primarily focused on the analysis of alkaloids, flavonoids, and terpenoids as key metabolites often used in chemotaxonomic studies [80,81]. However, this technique is equally efficient for essential oils. For example, in investigations focusing on the family Lamiaceae, LC-MS-Qtof has been effectively used for the chemotaxonomical profiling of essential oils, flavonoids, and phenolic compounds [82,83].

Similarly, in *Citrus* species (Rutaceae), LC-MS-Qtof has been used to distinguish between species based on terpenoid profiles, with particular attention paid to limonene, a characteristic compound of these species [84]. Likewise, in the Solanaceae family, LC-MS-Qtof has been extensively used to analyze bioactive alkaloids like capsaicin and nicotine [85]. In an investigation, LC-MS-Qtof was used to study *Capsicum annuum* (chili pepper)***,*** with capsaicinoids identified as key chemotaxonomic markers for this species [86]. Similarly, in the Asteraceae family, LC-MS-Qtof has proven valuable for identifying bioactive compounds in *Artemisia* (wormwood) and *Echinacea* species, both of which are widely used in traditional medicine. For example, *A. annua* is very well known for its antimalarial properties, which rely on the presence of artemisinin. It has been studied extensively using LC-MS-Qtof to determine its sesquiterpene content [87], and *E. purpurea*, which is known for its immune-boosting properties, has been analyzed to characterize its contents of echinacoside and other caffeic acid derivatives [88]. These examples highlight the increasing use of LC-MS-Qtof for the validation and authentication of medicinal plants. As with other analytical methods, the coupling of LC-MS-Qtof data with PCA and CA is gaining the attention of researchers for its ability to make data analysis more efficient and interpretation more reliable [89]. Modern LC-MS platforms leverage extensive mass spectral libraries to enable rapid and accurate compound identification by matching acquired spectra against reference databases. Prominent libraries include the comprehensive NIST Mass Spectral Library; METLIN, which specializes in metabolites and natural products, the open-access MassBank repository; and the commercial high-resolution mzCloud database with predictive fragmentation features [90,91,92]. Collaborative platforms like GNPS facilitate community-driven annotation of MS/MS data, especially for natural products [93]. Integrated software tools, such as Thermo Fisher’s Compound Discoverer, Agilent’s MassHunter, Waters’ UNIFI, and Sciex OS [93], utilize these libraries for automated spectral matching, molecular formula prediction, and in silico fragmentation, significantly improving identification confidence for complex mixtures.

### 6.4. MALDI-TOF MS

Matrix-assisted laser desorption/ionization time-of-flight mass spectrometry has become a key technique in chemotaxonomy [94]. The MALDI-TOF MS system employs a matrix to assist in the ionization of molecules, which are then analyzed using time of flight to identify their characteristic mass-to-charge ratios (*m*/*z*). This technique’s ability to generate unique spectral fingerprints of an organism’s metabolites, particularly proteins and lipids, has made MALDI-TOF MS invaluable in taxonomic studies [95].

As a recent example, MALDI-TOF MS was utilized to identify alkaloids and other bioactive compounds in species of *Cucurbita* (squash plants), which are used in traditional medicines [96]. By analyzing the protein and lipid profiles, researchers were able to categorize these species more accurately than when using traditional morphological approaches. In addition, the MALDI-TOF MS analysis revealed the presence of several important bioactive compounds, including cucurbitacins (e.g., cucurbitacin B and cucurbitacin E), quinolone alkaloids, and flavonoids, as well as phenolic acids like caffeic acid and chlorogenic acid [96]. Similarly MALDI-TOF MS analysis provided a comprehensive chemical profiling of closely related species and strains of *Echinacea*, including *E. purpurea* and *Echinacea angustifolia*, that highlighted common chemical compounds [97]. This analysis is crucial in ensuring the correct species is used in medicinal products, as different species may have different active compounds that contribute to immune modulation.

### 6.5. Nuclear Magnatic Resoanace (NMR)

Nuclear magnetic resonance spectroscopy offers a non-destructive, detailed method for characterizing the structures of plant metabolites and is especially useful for those not easily characterized via other methods [98]. It provides extensive information about the chemical environment of atoms within a molecule, allowing for the identification of complex compounds with high specificity [99]. It is important to note that NMR is often used in conjunction with other techniques, such as HPLC (Preparative), GC-MS, LC-MS, or LC-MS-Qtof. In such combinations, NMR can greatly enhance plant identification in families with diverse metabolites. For instance, in the Apiaceae family, for which flavonoids and coumarins are essential secondary metabolites, studies on *Angelica* and *Coriandrum* species have successfully employed NMR to elucidate unique flavonoid, glycoside, and furanocoumarin profiles that are critical for chemotaxonomic classification within these genera [100,101]. Such metabolite profiles can be directly correlated with a plant’s therapeutic properties, not only allowing accurate species identification but also effective quality control [102]. Similarly, NMR spectroscopy has greatly facilitated chemotaxonomy in the Lamiaceae family [20,103], where it has been extensively used to profile terpenoids, such as menthol, carvacrol, and eugenol, which serve as chemical markers [104] for distinguishing between closely related species. For instance, *M. piperita* and *Mentha spicata* (spearmint) can be differentiated based on their distinct terpenoid profiles, as revealed through NMR analysis [105]. Overall, the phytochemistry and plant science literature is replete with NMR analyses, and no phytochemical research is complete without one.

## 7. Current Trends in the Identification of Medicinal Plants

**Figure 3:**
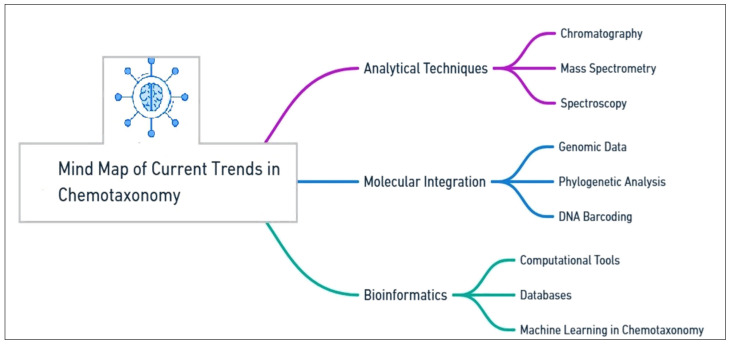
An overview of current trends in chemotaxonomy.

In recent years, advancements in analytical and molecular technologies have significantly enhanced the scope and precision of chemotaxonomic studies. Furthermore, the integration of chemotaxonomy with bioinformatics and computational tools has streamlined data analysis, enabling more comprehensive studies of plant species and their medicinal properties. In this section, we review current trends in chemotaxonomy, focusing on the integration of molecular tools and multivariate analyses and the computational and technological advancements that are pushing the field forward (Figure 3)**.**

### 7.1. Integrating Molecular Techniques with Chemotaxonomy

Molecular techniques complement traditional chemotaxonomy analyses well, and recent advancements are enabling even more accurate identification and classification of medicinal plants. Two molecular approaches, DNA barcoding and metabolomics, have gained significant attention [24,106]. By integrating chemical and molecular information, plant identification can be more authentic.

#### 7.1.1. DNA Barcoding

DNA barcoding has emerged as a critical tool in modern plant taxonomy, as it provides a rapid and reliable method for plant species identification. By utilizing the DNA sequences of short, universally accepted genomic regions, such as *rbcL* and *matK* gene regions and the ITS (internal transcribed spacer) region of the ribosomal RNA gene [107], DNA barcoding enables precise, species-level identification, even for morphologically similar species or when only incomplete plant specimens are available [108]. These regions are selected due to the balance between conservation and variation their sequences exhibit, which allows differentiation among species while maintaining a high degree of genetic stability across plant families [109]. By helping to catalog species, DNA barcoding aids in the conservation of plant biodiversity, especially in regions with rich but under-studied flora [110]. Barcoding, along with the computational tools and global genomic databases that have grown in its wake, has enabled researchers to address challenges related to species identification with enhanced efficiency and accuracy, driving significant advancements in plant biology and botanical research.

#### 7.1.2. Metabolomics

Metabolomics, the comprehensive study of metabolites in organisms, has emerged as a powerful tool in chemotaxonomy [24]. For medicinal plants, this approach involves analyzing the complete set of metabolites in a plant, including both primary and secondary metabolites, which are critical for identification. By employing techniques like HPLC, GC-MS, and NMR, metabolomics enables the profiling of plant species based on their metabolic signatures [111]. The integration of metabolomics into chemotaxonomy provides a deeper understanding of the functional roles of plant metabolites in medicinal efficacy, enhancing the identification of bioactive compounds and improving the overall quality control of medicinal plant products [20].

### 7.2. Multivariate Analysis in Chemotaxonomy

Multivariate analyses are crucial for processing and interpreting the large datasets generated by many chemotaxonomic techniques. Principal component analysis and CA are two commonly used methods that help researchers extract meaningful patterns and relationships from complex chemical datasets, facilitating plant identification and classification [44,112].

#### 7.2.1. Principal Component Analyses (PCA)

Principal component analyses are widely employed in chemotaxonomy to reduce the dimensionality of large chemical datasets while retaining the most significant variance [113]. By transforming the data into a set of orthogonal components, PCA facilitates the identification of patterns and trends that may not be immediately apparent in the raw data [17]. In the context of medicinal plants, PCA helps in grouping plant species based on their chemical profiles, providing insights into the chemical diversity of plant families or genera. This technique is particularly useful when dealing with large datasets generated when combining multiple analytical techniques, such as HPLC, GC-MS, and NMR [114].

#### 7.2.2. Cluster Analysis (CA)

Cluster analysis (CA) complements PCA by grouping plant species or samples based on similarities in their chemical profiles. This method uses algorithms, such as hierarchical clustering or k-means clustering, to classify plant species into distinct groups or clusters [115]. It is particularly valuable when dealing with closely related species or varieties that exhibit overlapping chemical traits, as it can identify subtle yet significant chemical differences [116]. By organizing species into clusters based on their chemical characteristics, CA not only aids in the identification of novel plant species but also enhances our understanding of the ecological adaptations of different plant groups and the evolutionary relationships within and between groups [117]. Overall, CA offers chemotaxonomy a powerful tool for the classification and identification of plant species, facilitating the discovery of bioactive compounds and improving the quality control of medicinal plant products.

## 8. The Role of AI in Chemotaxonomy

**Figure 4:**
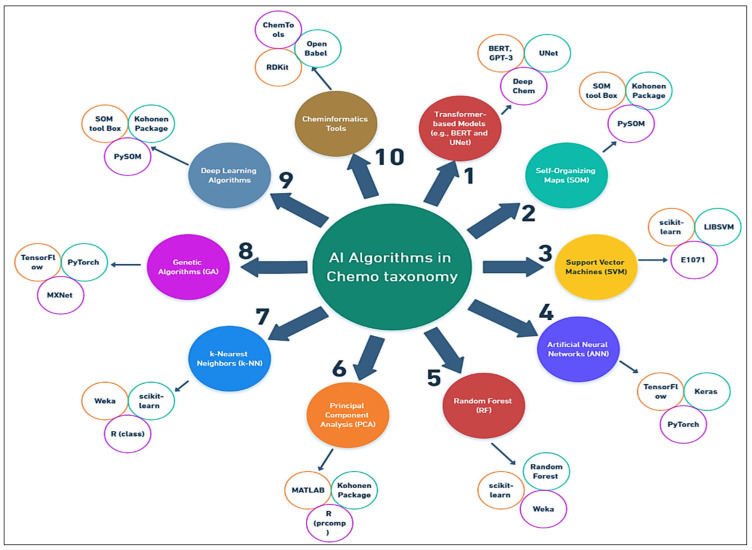
Potential of AI tools in chemotaxonomical identification of medicinal plants.

Artificial intelligence has emerged as a transformative force in chemotaxonomy, where it is used to enhance plant identification, classification, and the discovery of bioactive compounds [20,118]. In particular, AI models based on ML, deep learning, and natural language processing offer powerful tools for processing and analyzing the vast chemical datasets generated by analytical techniques like NMR, GC-MS, and LC-MS-Qtof [119]. Furthermore, AI can assist in automating plant identification, improving pattern recognition among chemical profiles, and enabling predictive analyses of plant compound bioactivity (Figure 4). In this section, we discuss the key areas where AI is making an impact in chemotaxonomy.

### 8.1. Data Analysis and Pattern Recognition

One of the primary applications of AI in chemotaxonomy is in the analysis of complex datasets through techniques like PCA and CA, as discussed earlier.

### 8.2. Automation of Plant Identification

Various AI models, especially deep learning algorithms like convolutional neural networks (CNNs), are increasingly used to automate plant species identification [120]. By training AI systems on large datasets of chemical fingerprints, these models learn to recognize specific metabolite patterns that distinguish one species from another [121]. The advantage of AI in this context is its ability to process data much faster than human experts and to handle larger datasets, leading to a more efficient identification process. For instance, CNNs have been applied to GC-MS and LC-MS-Qtof data to automate the classification of plant species [122]. These AI models are able to detect chemical signatures in chemical profiles containing secondary metabolites, such as terpenoids, flavonoids, and alkaloids, which are characteristic of specific plant families [123].

### 8.3. Integration of Multi-Omics Data

Given their ability to discern patterns in large datasets, AI models are playing a significant role in the integration of various omics data types (e.g., genomics, metabolomics, and environmental data), facilitating more accurate and comprehensive plant identification and classification [124,125]. By efficiently combining metabolomics data from LC-MS-Qtof and GC-MS analyses with genomic data, AI models can provide more robust predictions about plant species and their bioactive compounds [126]. This integration helps enhance plant chemotaxonomy by offering a more holistic view of a plant’s chemical, genetic, and environmental profile.

### 8.4. Predicting Bioactivity and Medicinal Potential

Artificial intelligence has significantly advanced the prediction of bioactivity, and thus medicinal potential, by allowing the analysis of extensive datasets to identify novel drug candidates and forecast their interactions and efficacy [127,128]. Researchers have been working to expedite drug discovery through machine and deep learning techniques, focusing on the prediction of protein structure, drug–target interactions, and molecular properties [129]. Similarly, AI models can more efficiently predict drug toxicity, bioactivity, and physicochemical properties, thereby streamlining the drug development process [130]. For example, in the evaluation of medicinal plants, AI algorithms have been trained to predict the anti-inflammatory, antimicrobial, and anticancer properties of plant compounds based on their chemical structure [131]. These AI-based methods can drastically reduce the time and resources required for experimental testing by providing preliminary insights into the therapeutic potential of specific compounds.

### 8.5. Advancements in AI Algorithms and Chemotaxonomy

Recent advancements in AI, particularly in deep learning and natural language processing, have further enhanced its application in chemotaxonomy. Newer algorithms are able to handle larger and more complex datasets, enabling faster and more accurate plant species identification based on chemical profiles [132]. For instance, transformer-based models like BERT (bidirectional encoder representations from transformers) have been applied to metabolomic data, improving our understanding of plant metabolites and their role in species differentiation [133,134]

## 9. Limitations of Chemotaxonomy

Despite its valuable contributions to the identification and classification of medicinal plants, chemotaxonomy faces several limitations that must be addressed before its full potential can be realized (Table 4). This section discusses several important points that must be considered.

### 9.1. Variability in Secondary Metabolite Profiles

Secondary metabolites in plants are highly influenced by environmental factors, such as soil conditions, climate, altitude, and seasonal changes, as well as genetic diversity [135]. This variability can lead to significant differences in alkaloid, terpenoid, and flavonoid profiles across regions or growing conditions, which complicates chemotaxonomic identification and classification [139].

### 9.2. Standardization Issues

A lack of standardized methods for analyzing plant chemical profiles presents another significant challenge in chemotaxonomy [135]. Different researchers may use different analytical techniques, such as HPLC, GC-MS, or NMR, under varying experimental conditions, which can lead to inconsistent results [140]. Additionally, the way plant extracts are prepared, including the solvents used and the extraction methods applied, can influence the chemical composition of the samples [141].

### 9.3. Lack of Comprehensive Databases

One unavoidable issue in chemotaxonomy is the absence of comprehensive and accessible databases that house detailed chemical profiles of medicinal plants [135]. While several databases have been developed to catalog plant species and their associated chemical compounds, these resources are incomplete, lack uniformity, or are not easily accessible [142]. This limits the utility of these databases for taxonomic and medicinal plant identification.

### 9.4. Accessibility and High Costs of Analytical Techniques

The high costs associated with advanced analytical techniques like HPLC, GC-MS, NMR, and LC-MS-Qtof are significant barriers to the widespread use of chemotaxonomy, especially in resource-limited settings [17]. The purchase and maintenance costs of the sophisticated equipment required for these techniques, as well as the cost of reagents and consumables, can be prohibitive for many research institutions and laboratories, particularly in developing countries.

### 9.5. Ethnobotanical Knowledge

Another limitation of chemotaxonomy is a lack of comprehensive ethnobotanical knowledge, which could inform the classification and identification of medicinal plants [135]. Ethnobotanical data, which includes knowledge of traditional plant usage in indigenous communities, is crucial for understanding the medicinal properties of plants and their modern applications [143]. However, much of this knowledge has not been systematically documented and is at risk of being lost as traditional practices fade away.

## 10. Challenges in Chemotaxonomic Identification

Despite the importance of chemotaxonomy, several limitations do also exist. For instance, there is high variability in plant chemical compositions, which can be influenced by various factors, including environmental conditions, developmental stage, and genetics [29,144]. This variability can complicate identification and classification, leading to inconsistencies in chemotaxonomic results [145]. Another challenge is the high cost and complexity of the analytical techniques required for identifying plant metabolites [146]. Similarly, a lack of standardized methods for using these techniques creates an issue [147]. Moreover, there is a deficiency in globally harmonized chemotaxonomic reference databases, limiting comparative analysis across species or genera. These technical disparities hinder the development of universal chemotaxonomic frameworks, underscoring the need for method standardization, data sharing platforms, and the integration of AI to streamline identification and interpretation.

## 11. Future Directions in Chemotaxonomy

As new tools and methodologies are emerging, chemotaxonomy is becoming an even more integral part of medicinal plant research and applications. This section highlights several key areas we believe should be considered important focuses of future research.

### 11.1. Integration of Multi-Omics Approaches

The convergence of genomics, transcriptomics, proteomics, and metabolomics is revolutionizing chemotaxonomy. Tools like MEGA, Cytoscape, and WGCNA enable pathway prediction and co-expression analysis of plant chemical components [148]. Similarly, databases such as MetaboLights, HMDB, and KEGG provide metabolite–gene linkage data that can assist in taxonomic discrimination [149]. Serval multi-omics workflows have recently been applied to cyanobacteria and algae, demonstrating the potential to open new paths in chemotaxonomy [150], which in fact adds to its relevance.

### 11.2. Application of AI and ML

Both AI and ML models are consistently being used to classify plants based on high-dimensional chemical fingerprints. Random forests and support vector machines have already been used in LC-MS-based medicinal plant classification [151]. Similarly, deep learning-based bioinformatics tools, such as DeepChem and DeepMetabolome, can process large metabolomics datasets for automated taxonomic classification [152].

### 11.3. Development of Comprehensive Chemotaxonomic Databases

Given the increasing importance of chemotaxonomy, there exists a great need to develop interoperable and centralized platforms that integrate genomics, phytochemistry, and environmental metadata. Databases like KNApSAcK, PlantCyc, MassBank, and NPASS can be used as foundations to create these resources [153].

### 11.4. Digital Herbarium Platforms with Integrated Chemoprofiling

Future digital herbaria could integrate high-resolution images, geospatial metadata, and chemical fingerprints from NMR, LC-MS, GC-MS, etc., enabling greater taxonomic and biochemical insight [154]. Current systems, like GBIF and iDigBio, lack chemical metadata, which can be helpful in this case [155]. Digital herbaria should allow spectral searchability, further enhancing the synergy created by the integration of data types.

### 11.5. Synthetic Biology for Metabolic Pathway Validation

Synthetic biology allows scientists to validate biosynthetic pathways inferred from chemotaxonomic studies [156]. By expressing gene clusters in model organisms (e.g., *Nicotiana benthamiana*’s secondary metabolites expressed in yeast), the origin and regulation of secondary metabolites can be studied [157]. This approach is crucial for confirming gene function, regulatory sequences, and enzyme interactions that are otherwise obscured in native plant systems due to redundancy or low expression levels [158].

### 11.6. Chemotaxonomy in Conservation and Drug Discovery

**Figure 5:**
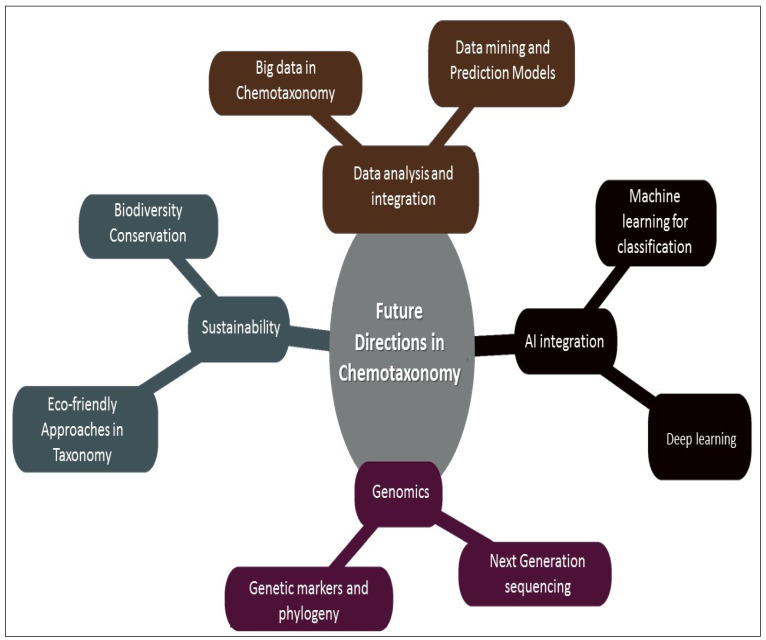
Future directions in chemotaxonomical identification of medicinal plants.

By linking phytochemical diversity with taxonomic and phylogenetic information, chemotaxonomy can be a very helpful tool for conservation biology and drug discovery [27]. By identifying taxa with rich and unique metabolite profiles, chemotaxonomy can guide conservation efforts toward chemically and evolutionarily valuable species, many of which may currently be endangered or underexplored [139]. This approach enhances the efficiency of bioprospecting, as taxonomic proximity to known medicinal plants often predicts similar bioactivity profiles. For instance, the chemotaxonomic mapping of phytochemical “hotspots” in the plant kingdom has been proposed as a way to identify priorities for both biodiversity protection and pharmaceutical exploration [159] (Figure 5).

## 12. Conclusions

Chemotaxonomy serves as a vital tool in medicinal plant research by enabling precise species identification and medicinal product authentication through the chemical profiling of secondary metabolites. Analytical techniques such as HPLC, GC-MS, NMR, LC-MS/QToF, and Fourier transform infrared spectroscopy are instrumental in detecting bioactive compounds, supporting both quality control and therapeutic agent discovery. When integrated with genomics and proteomics, chemotaxonomic approaches facilitate the elucidation of biosynthetic pathways, enhancing our understanding of metabolite diversity and function. Such methodologies are particularly critical for ensuring the safety and efficacy of herbal medicines by preventing misidentification and adulteration. Moreover, chemotaxonomy contributes to drug development by uncovering novel phytochemicals with pharmacological potential. Emerging trends in this field involve the incorporation of AI and ML to accelerate compound identification and classification. Despite these advancements, the standardization of analytical protocols and the development of comprehensive global databases remain key challenges. Continued progress in chemotaxonomy is expected to drive innovations in plant-based drug discovery and personalized phytotherapeutics and accelerate traditional medicine validation while promoting sustainable utilization of botanical resources.

## Figures and Tables

**Table 1:** Primary and secondary metabolites and their role in plant health.

Metabolite Type	Metabolite Class	Occurrence in Plant Part	Metabolite Role	References
Primary metabolite	Carbohydrates	Leaves	Energy source, essential for respiration.	[30,36]
Primary metabolite	Amino Acids	Leaves, Roots	Building blocks of proteins, crucial for plant growth.	[30,36]
Primary metabolite	Fatty Acids	Seeds, Leaves	Vital for membrane structure and energy storage.	[30,36]
Primary metabolite	Chlorophyll	Leaves	Key for photosynthesis, converting light into energy.	[30,36]
Secondary metabolite	Alkaloids	Roots, Seeds	Defensive compounds, deter herbivores and pathogens.	[37,38]
Secondary metabolite	Flavonoids	Flowers, Leaves	Provide UV protection, antioxidant properties, and pigmentation.	[37,38]
Secondary metabolite	Terpenoids	Leaves, Roots	Involved in plant defense.	[37,38]
Secondary metabolite	Phenolics, Tannins	Roots, Leaves	Play roles in defense, antioxidation, and stress response.	[37,38]

**Table 2:** A comparative review of morphological and chemotaxonomical identification of medicinal plants.

Feature	Morphological Identification	Chemotaxonomical Identification	Citations
Basis of classification	Observable physical traits (leaf shape, flower structure, stem, etc.)	Chemical composition, mainly secondary metabolites and other biochemical markers	[44,45]
Attributes for examination	External features (e.g., leaves, flowers)	Secondary metabolites and primary compounds	[44,46]
Ecological impact	High (traits may vary due to climate, soil, etc.)	Low (compounds are more stable)	
Tools required	Microscope, visual inspection	Chromatography, spectroscopy	[44,46]
Resolution	precise due to phenotypic Often limited at intraspecific level (varieties, subspecies)	Can distinguish species and intraspecific taxa	[44,45,46]
Part used	Leaf shape, flower color, stem structure	Alkaloids, flavonoids, composition of plant part (terpenoids, amino acids)	[44,46]
Use in modern taxonomy	Foundational, and widely used in conjunction with molecular methods	Widely used in conjunction with molecular methods	[44,46]
Speed and accessibility	Relatively quick and low cost, can be carried out in the field	More time-consuming and costly, requires laboratory equipment	[44,45,46]
Cryptic species	Phenotype plasticity Difficulties in identification of cryptic Species	Requires specialized equipment and expertise More effective; can detect biochemical differences in cryptic species	[44,46]

**Table 3:** Various analytical techniques used in chemotaxonomy.

Analytical Technique	Typical Uses	Accuracy/Precision	Types of Secondary Metabolites	Citations
UV-Vis Spectroscopy	Quantification	Moderate accuracy, ideal for fast and non-destructive quantification.	Flavonoids, phenolic compounds, carotenoids, alkaloids	[51]
FTIR	Identification of functional groups and molecular structures	Good resolution for functional group identification. Lower sensitivity compared to MS-based techniques.	Terpenoids, alkaloids, flavonoids, phenolic acids, lipids	[52]
HPLC	Separation and quantification of compounds, particularly in mixtures	High accuracy in separating complex mixtures. Precision depends on column and mobile phase.	Alkaloids, flavonoids, phenolic acids, glycosides, terpenoids	[53]
GCMS	Identifying and quantifying volatile compounds, especially in complex mixtures	High sensitivity and precision for volatile organic compounds, good for trace analysis.	Volatile terpenes, essential oils, fatty acids, aldehydes	[53,54]
LCMS-QTOF	Comprehensive profiling of metabolites and complex biomolecules	Very high sensitivity and accuracy, capable of accurate molecular mass determination, used for complex samples.	Alkaloids, flavonoids, peptides, lipids, steroids, phenolic compounds	[53,55]
MALDI-TOF MS	High-throughput analysis of biomolecules, especially proteins and peptides	High sensitivity for large biomolecules like proteins, peptides, and lipids. Excellent for high-throughput applications.	Peptides, proteins, lipids, alkaloids	[53,55]
NMR	Structural elucidation, identification of compounds, and quantification in small to medium-sized molecules	High accuracy for molecular structure determination. Limited sensitivity compared to MS techniques, but excellent for structural analysis.	Alkaloids, flavonoids, terpenoids, phenolic compounds, saponins	[53]

**Table 4:** Limitations of chemotaxonomy with possible alternate solutions.

Limitation	Description	Possible Alternate	Citations
Variability in Secondary Metabolite Profiles	Variability due to environmental, genetic, or developmental factors.	Use DNA barcoding or meta barcoding as an alternative.	[135]
Standardization Issues	No standardized methodology for metabolite analysis.	Standardize techniques like mass spectrometry or NMR.	[135,136]
Lack of Comprehensive Databases	Chemotaxonomic databases are often incomplete.	Collaborate to build comprehensive chemotaxonomic databases.	[135,137]
Accessibility and High Costs of Analytical Techniques	High costs and specialized expertise needed for advanced techniques.	Utilize portable, low-cost devices for on-site analysis.	[135]
Challenges in Chemotaxonomic Identification	Overlapping chemical profiles make accurate identification difficult.	Use multi-omics approaches for more accurate identification.	[27,135,138]

## Data Availability

Data are contained within the article.

## Supplementary Materials

The following supporting information can be downloaded at: https://www.mdpi.com/article/10.3390/plants14142234/s1, Figure S1. Structure of diverse secondary metabolites (and their classes) commonly found in plants. Figure S2. Overview of plant secondary metabolites in correlation with plant identification.
