# Supplementary material for: Chemotaxonomy, an Efficient Tool for Medicinal Plant Identification: Current Trends and Limitations
Source: Plants (Basel). 2025 Jul 19;14(14):2234. doi: 10.3390/plants14142234 (PMC12299962; doi:10.3390/plants14142234)
Supplement: Supplementary file 1 [file plants-14-02234-s001.zip › plants-3697169-supplementary.pdf]

Review

Chemotaxonomy, an Efficient Tool for Medicinal Plant Identification: Current Trends and Limitations

Adnan Amin and SeonJoo Park *

Department of Life Sciences, Yeungnam University, Gyeongsan 38541, Republic of Korea; adnan.amin@yu.ac.kr
* Correspondence: sjpark01@ynu.ac.kr

Supplementary data

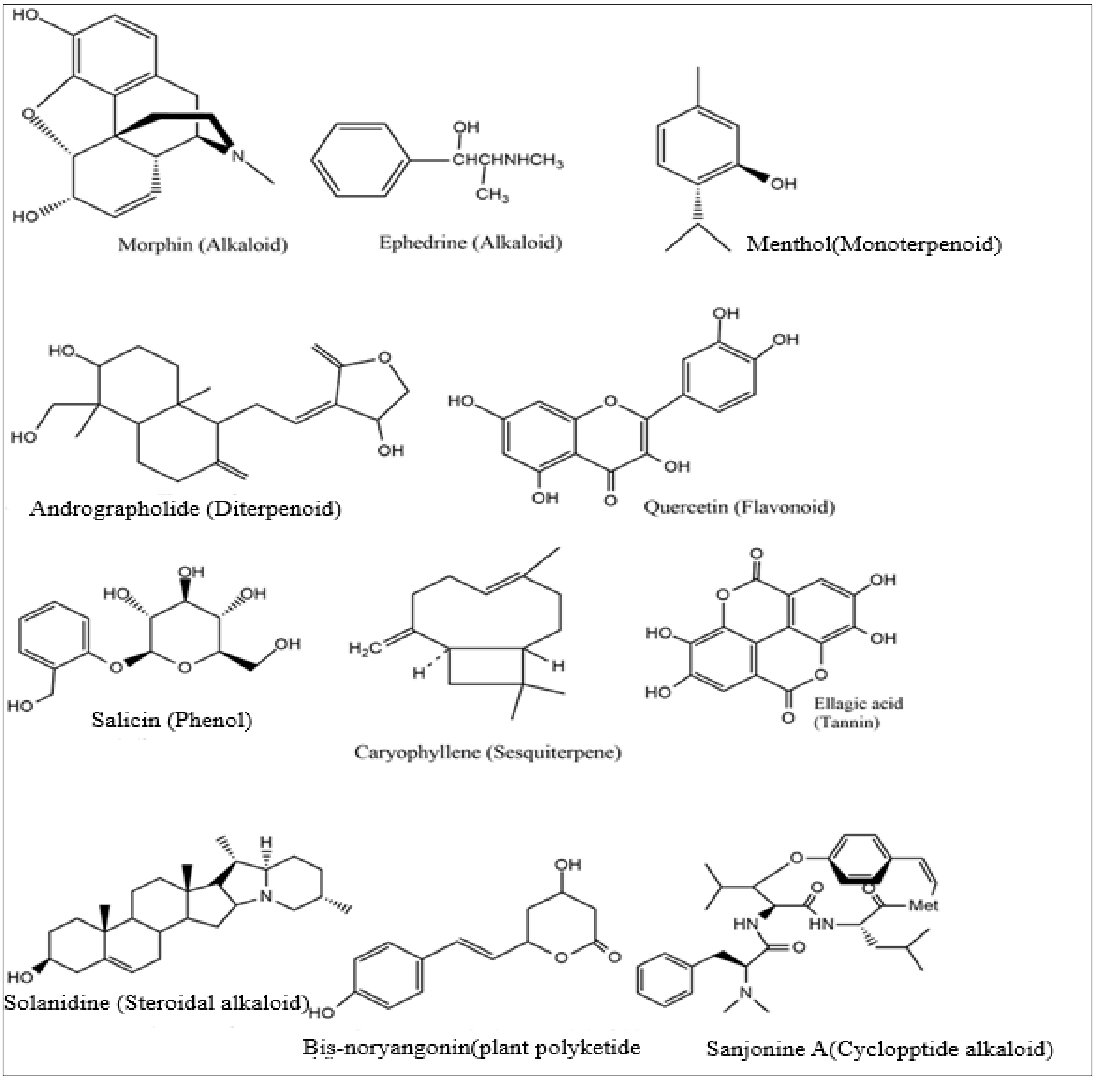

Figure S1. Structure of diverse secondary metabolites (and their classes) commonly found in plants.

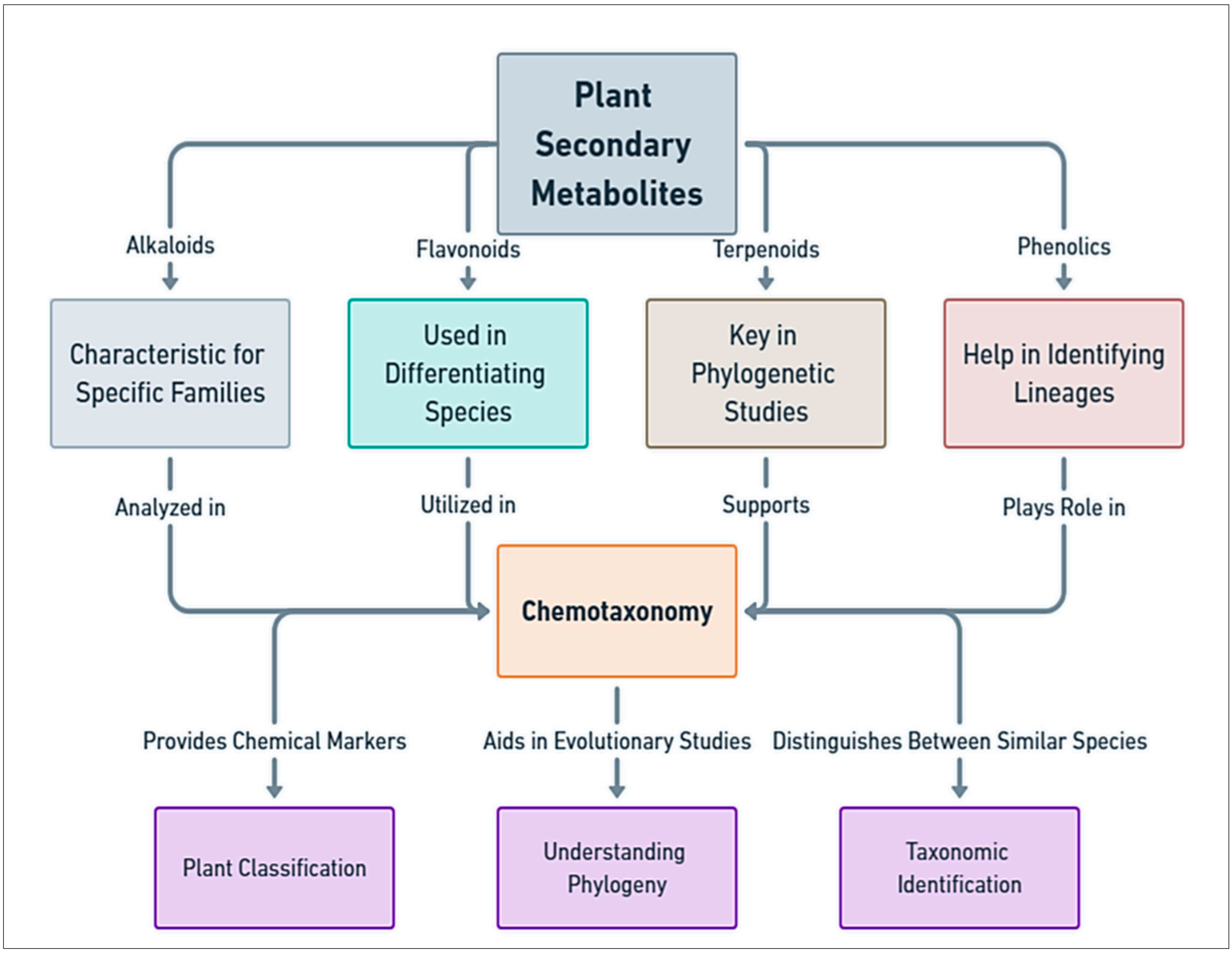

Figure S2. Overview of plant secondary metabolites in correlation with plant identification.
